# First Whole Genome Sequencing of Crimean–Congo Hemorrhagic Fever Virus (CCHFV) in Tick Species within Ghana

**DOI:** 10.1155/2023/2063317

**Published:** 2023-08-18

**Authors:** Ronald Essah Bentil, Seth Offei Addo, Mba-tihssommah Mosore, Selassie Kumordjie, Clara Yeboah, Bright Agbodzi, Eric Behene, Janice Tagoe, Bernice Olivia Ama Baako, Victor Asoala, Richard Osei Ampadu, Daniel Lartei Mingle, Edward O. Nyarko, Anne T. Fox, Andrew G. Letizia, Joseph William Diclaro, Terrel Sanders, Daniel Oduro, Shirley C. Nimo-Paintsil, James Harwood, Samuel Kweku Dadzie

**Affiliations:** ^1^Parasitology Department, Noguchi Memorial Institute for Medical Research, College of Health Sciences, University of Ghana, Legon, Accra, Ghana; ^2^Navrongo Health Research Centre, Navrongo, Upper East Region, Ghana; ^3^Ghana Armed Forces Veterinary Service, Accra, Ghana; ^4^Public Health Division, 37 Military Hospital, Ghana Armed Forces Medical Service, Accra, Ghana; ^5^U.S. Naval Medical Research Unit No. 3, Ghana Detachment, Accra, Ghana; ^6^Infectious Diseases Directorate, Naval Medical Research Center, Silver Spring, MD, USA; ^7^Navy Entomology Center for Excellence, Jacksonville, FL, USA; ^8^Immunology Department, Noguchi Memorial Institute for Medical Research, College of Health Sciences, University of Ghana, Legon, Accra, Ghana; ^9^U.S. Naval Medical Research Unit No. 3, Sigonella, Italy

## Abstract

Crimean–Congo hemorrhagic fever (CCHF) is a serious viral zoonotic disease spread by ticks and caused by the Crimean–Congo hemorrhagic fever virus (CCHFV). The emergence and reemergence of CCHF in various nations in the Eastern Mediterranean Region over the last decade have shown a growing risk of the disease spreading to new areas, especially in population-dense and livestock trade-dominant areas. There is a lack of updated information on the risk of CCHFV in the Greater Accra and Upper East Regions of Ghana. Due to the paucity of available data, this study sought to identify the tick species diversity in Ghana and to ascertain the CCHFV strains they may carry. A total of 705 ticks were collected from 188 cattle and 11 horses and morphologically identified. Three tick genera (*Hyalomma*, *Amblyomma*, and *Rhipicephalus*) were observed, with the predominant species being *Hyalomma rufipes* (*n* = 290, 41.1%). The CCHFV infection rates of 0.78%, 0.69%, and 0.64% were recorded in *Hyalomma truncatum*, *H. rufipes*, and *Amblyomma variegatum*, respectively. No infection was detected in the *Rhipicephalus* species. Furthermore, a strain was successfully recovered using next-generation sequencing. The strain belongs to genotype 3 and shared 98.9% nucleotide identity with DQ211641_Mauritania_1984 and MF287636_Spain_2016. Findings from this study suggest the possible importation of the virus into the country through trade, and potentially, a public health threat to humans who may have primary contact with livestock.

## 1. Introduction

In several countries across sub-Saharan Africa, epidemiological data on tick prevalence and distribution, as well as tick-borne diseases, are largely unknown [1]. The absence of information, possibly due to unavailable data or underreporting of available data, may be an indication that the risk these diseases pose to human health in the region is not accurately estimated. The difficulty in identifying tick species, managing populations, as well as diagnosing and treating infections of tick-borne pathogens makes efforts to combat these emerging diseases challenging in West Africa [1, 2].

The Crimean–Congo hemorrhagic fever virus (CCHFV) is transmitted to humans from bites of infected ticks, crushing infected ticks near an open wound, contact with blood or tissues from infected patients and animals, and consuming unpasteurized milk from infected animals [3]. Crimean–Congo hemorrhagic fever (CCHF) causes fatal hemorrhagic infections in humans [4], and in Ghana, ticks of the genera *Hyalomma* and *Amblyomma* have been implicated in transmission [5]. Reports indicate that CCHFV is transmitted within tick populations during cofeeding and passed on through transstadial and transovarial transmission and that infection is lifelong [6–8].

Livestock such as cattle, sheep, and goats serve as amplifying hosts for the virus [9]. With the high dependence on livestock for food in Ghana and the presence of suitable tick species, CCHFV is of great veterinary and public health importance [8]. The symptoms shown by an infected human include dizziness, severe headache, nausea, fever, cardiovascular and neuropsychiatric changes, diarrhea, and hemorrhages [10]. Furthermore, in humans, the case-to-fatality ratio varies between 5% and 80%, depending on the availability of appropriate medical care and the severity of clinical manifestations at the time of diagnosis [11, 12].

In Ghana, CCHFV has been reported to occur in ticks, and through serology, some abattoir workers have been identified to be exposed to the CCHFV due to their contact with infected ticks [5]. However, the prevalence and circulating strains of CCHFV are not known in Ghana. Therefore, to better inform Ghana's public health sector, this study sought to determine the presence of CCHFV in ticks collected within selected study sites. The findings from this study will be essential in formulating control measures to prevent infections in animal owners and handlers in Ghana.

## 2. Methodology

### 2.1. Study Area

**Figure 1:**
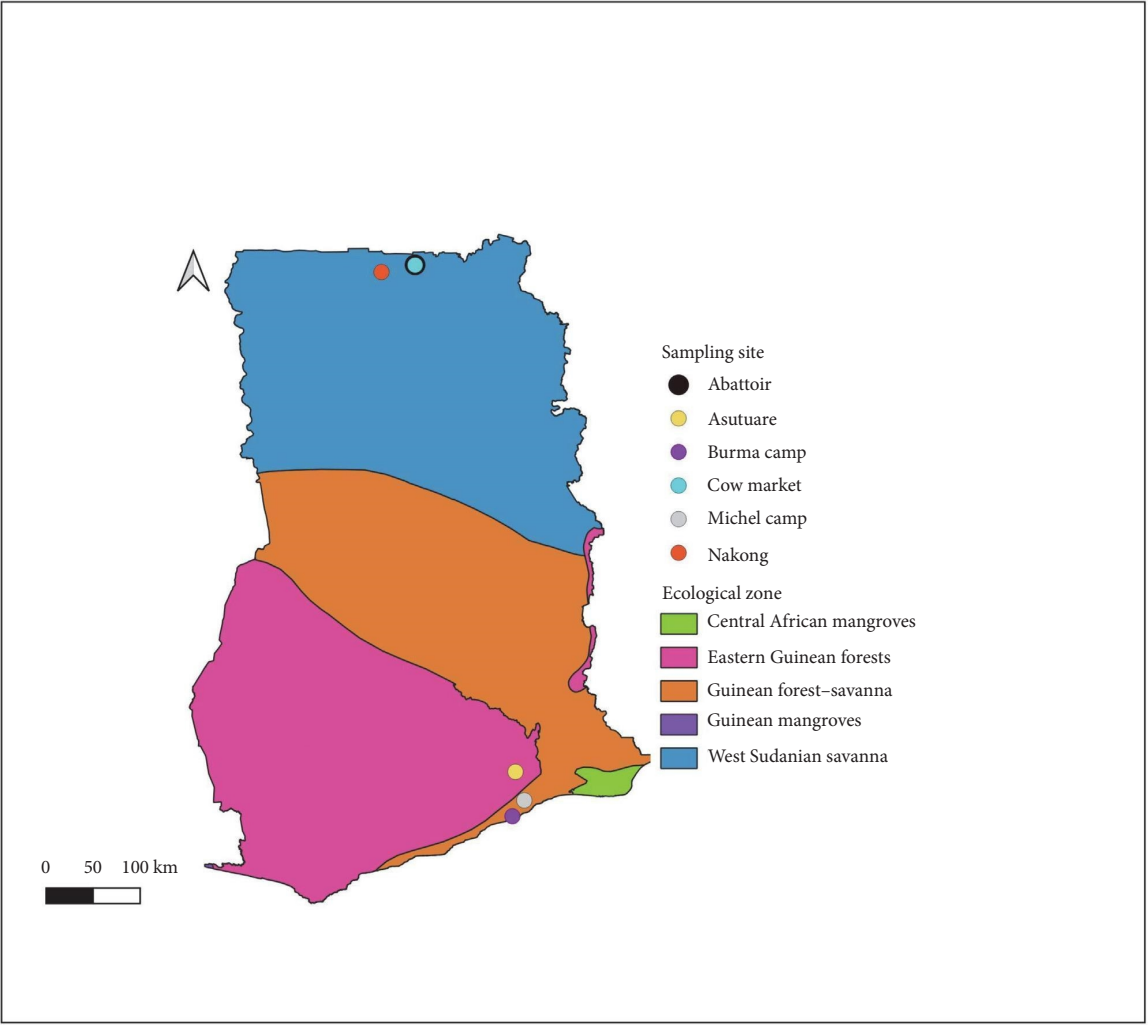
A map of Ghana showing sampling sites from the various ecological zones. The map was developed using the Quantum Geographic Information System (QGIS version 3.30.3-'s-Hertogenbosch). The ecoregion data layer was obtained from Terrestrial Ecoregions GIS Data—GIS Lounge.

Sampling was carried out within environs in the Upper East (Guinean mangroves and West Sudanian savanna) and Greater Accra (Central African mangroves, Eastern Guinean forests, Guinean forest-savanna) Regions. Within the two regions, the sampling sites were selected based on the collaborative efforts of the Ghana Armed Forces, the Navrongo Health Research Centre (NHRC), and the U.S. Naval Medical Research Unit No. 3 (US NAMRU-3). The sites included military-owned kraals at Burma camp (five infantry battalion and three mounted squadron), Michel camp (one infantry battalion), and Asutsuare Training camp within Greater Accra, as well as an abattoir, a cattle market, and Nakong community within Navrongo (Figure 1).

### 2.2. Tick Collection and Identification

A cross-sectional study was conducted from January to March 2020 in the selected study sites. Each animal was examined for tick infestation, and ticks present on each animal were collected using forceps. The ticks were placed into labeled tubes containing RNA Later and transported to the acute febrile illness laboratory at the Noguchi Memorial Institute for Medical Research for morphological identification using available taxonomic keys covering tick species of domestic animals in Africa [13].

### 2.3. Molecular Analysis of Ticks for CCHFV Genome

#### 2.3.1. RNA Extraction and Reverse Transcription-qPCR

Extraction of total nucleic acid from each tick was carried out using QIAamp Viral RNA Mini Kit (250) following the manufacturer's protocol. In screening for CCHFV, in-house reagents from the U.S. Army Medical Research Institute of Infectious Diseases (USAMRIID) were used with primers targeting the S segment of the CCHFV genome [14].

The total volume of the reaction was 20 *μ*l, comprising 14.6 *μ*l of prepared master mix, 0.4 *μ*l of platinum Taq, and 5 *μ*l of the template (nucleic acid extract). Polymerase chain reaction (PCR) was performed using the Applied Biosystems (USA) 7300 real-time PCR system with cycling conditions as follows: one cycle of the first hold for 15 min at 50°C, one cycle of the second hold for 5 min at 95°C, 45 cycles of the third hold for 1 s at 94°C, 30 s at 55°C (fluorescence read and data acquisition), for 5 s at 68°C and one cycle of the fourth hold at for 30 s at 40°C.

#### 2.3.2. Whole-Genome Sequencing

Sequencing libraries were prepared using the Illumina DNA prep with enrichment (Illumina Inc, San Diego, CA, USA) according to the manufacturer's instructions. Viral enrichment was performed using custom target capture probes (Twist Bioscience, San Francisco, CA, USA). The extracted RNA was fragmented, spiked with mosquito RNA to enhance ligation efficiency, and reverse-transcribed to cDNA. Spiking with mosquito RNA at a known concentration ensures amplification bias is corrected and can be used to normalize and quantify in silico the sequencing output [15, 16]. Dual indexing of cDNA libraries was achieved with the use of IDT unique dual indexes (IDT, Coralville, IA, USA). The libraries were enriched using the 1-plex pooling strategy following the protocol described by Blackley et al. [17]. Barcoded, pooled libraries were sequenced on an Illumina iSeq 100 system (Illumina, San Diego, CA, USA).

#### 2.3.3. Statistical Analysis

Descriptive statistics such as frequencies and percentages were used to determine tick species distribution and infection status. The *χ*^2^ test was used to determine the association between each tick species and region. Statistical significance was set at *p* < 0.05.

#### 2.3.4. Sequence Analysis

Demultiplexed raw FASTQ files were quality filtered to Phred score ≥20, filtered for a minimum read length of 20 bp, and adaptor trimmed using BBDuk (BBMap—Bushnell B.—sourceforge.net/projects/bbmap/). Read quality was confirmed using the FastQC tool.

The resultant high-quality reads were used for *de novo* assembly using the SPAdes assembler v 3.15.2. The resultant contigs were scaffolded against RefSeq sequences of the various segments (S: NC_005302.1; M: NC_005300.2; L: NC_005301.3). The consensus sequence for each segment was then used to query the nonredundant nucleotide database (GenBank) to obtain the best matching reference sequence (S: MF287636; M: MN689740; L: DQ211615). The retrieved reference was used for reference-based assembly using Bowtie2.

#### 2.3.5. Phylogenetic Analysis

Phylogenetic analysis was based on the S, M, and L segments. Sequences representing the major CCHFV genotypes retrieved from GenBank were analyzed alongside our strain. Sequences were aligned using MAFFT v1.5.0 implemented in Geneious Prime [18]. To correct for the effects of ambiguous alignments due to the polymorphisms in 5′ and 3′ untranslated regions, the sequences were trimmed to the open reading frames (ORFs), and all subsequent phylogenetic analyses were conducted on the ORFs. Phylogenetic model and tree inference were simultaneously conducted in IQ-TREE v 2.0.3, executing 1,000 ultrafast bootstraps [19]. Tree visualization and annotation were done with FigTree v1.4.4 [20].

## 3. Results

### 3.1. Tick Species Composition, Distribution, and CCHFV Infection Rates

A total of 705 ticks were collected from cattle (*n* = 188) and horses (*n* = 11). Three tick genera (*Hyalomma*, *Amblyomma*, and *Rhipicephalus*) were observed in the study. Approximately 0.6% of the ticks tested positive for CCHFV. The tick species diversity and CCHFV infection rates are presented in Table 1.

Tick species distribution of *Hyalomma rufipes*, *Rhipicephalus* spp., and *Rhipicephalus* (*Boophilus*) sp. were significantly different between the two sampled regions (Table 2).

### 3.2. CCHFV Prevalence in Ticks

Four of the 705 (0.6%) ticks screened were positive for CCHFV, which was detected in *Hyalomma truncatum* (0.78%), *Amblyomma variegatum* (0.64%) and *H. rufipes* (0.69) (Table 1). All ticks that tested positive for CCHFV were collected from the Upper East region.

### 3.3. Whole-Genome Sequencing

One sample (T-826_GHA/2021) with a ct value of 24.94 was successfully sequenced. A total of 341,202 reads were generated for T-826_GHA_2020 from the sequencing run. Out of these reads, 292,856 survived the initial filtering steps. After the reference-based assembly, 16,296, 5,867, and 41,274 reads were mapped to the S, M, and L segments, respectively. This resulted in the average sequencing depth of 1,368x, 181x, and 412x for the S, M, and L segments, respectively. The S, M, and L segments were recovered at coverage of 97.8%, 72%, and 98.8% with respect to the reference sequences.

The sequence obtained from this study has been deposited in GenBank as follows:

T-826_S_Ghana_2020 (OQ441063), T-826_M_Ghana_2020 (OQ441064), and T-826_L_Ghana_2020 (OQ441065).

### 3.4. Phylogenetic Analysis

**Figure 2:**
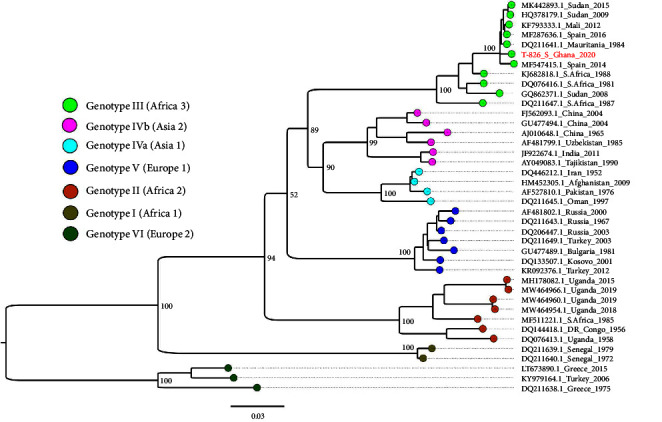
S segment maximum-likelihood phylogeny of CCHF viruses from different geographical origins alongside the Ghana strain (red text). Tip colors indicate geographical origins and are interpreted in the color key. Critical nodes are labeled with bootstrap values. The tree was visualized in FigTree.

**Figure 3:**
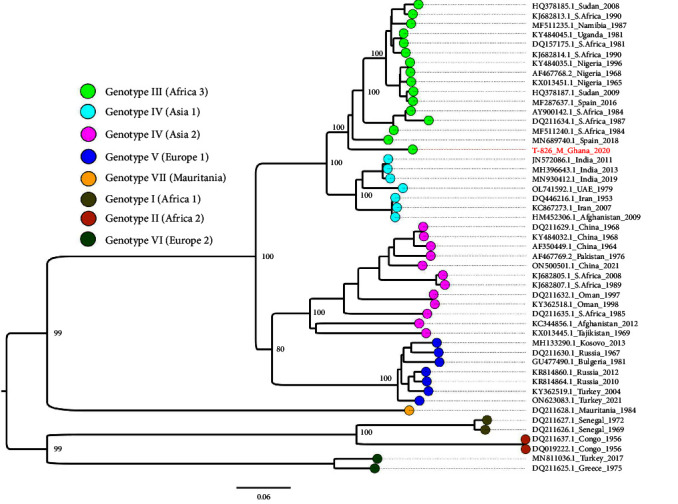
M segment maximum-likelihood phylogeny of CCHF viruses from different geographical origins alongside the Ghana strain (red text). Tip colors indicate geographical origins and are interpreted in the color key. Critical nodes are labeled with bootstrap values. The tree was visualized in FigTree.

**Figure 4:**
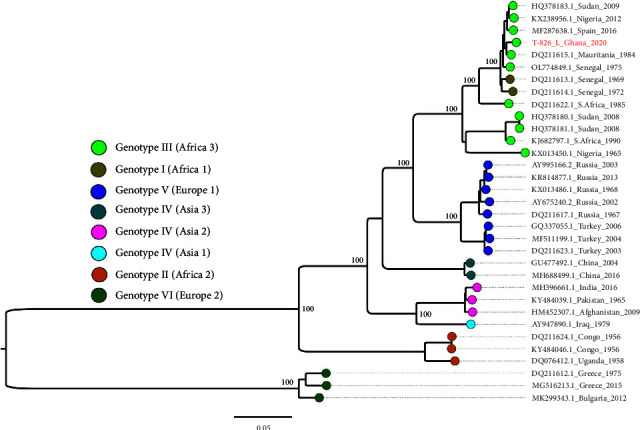
L segment maximum-likelihood phylogeny of CCHF viruses from different geographical origins alongside the Ghana strain (red text). Tip colors indicate geographical origins and are interpreted in the color key. Critical nodes are labeled with bootstrap values. The tree was visualized in FigTree.

Phylogenetic analysis of the S segment revealed that our strain clustered with other strains in the genotype III (Africa 3) group (Figure 2). Our strain was closest to a 2016 Spain strain (MF287636) with a nucleotide identity of 98.17% and ORF amino acid identity of 99.6%. The M segment clustered with genotype III (Africa 3) strains (Figure 3), with the closest GenBank match as a Spain 2018 strain (MF689740.1). The nucleotide identity between the two strains was 91.85, while the amino acid identity between the ORFs was 97.2%. The L segment clustered with genotype III (Africa 3) strains (Figure 4) and was closest to a 1984 Mauritania strain (DQ211615) with a nucleotide identity of 98.72%. The amino acid identity between the ORFs was 99.5%.

## 4. Discussion

*Hyalomma* ticks are considered to be the principal vectors of CCHFV [4, 8]. In this study, *Amblyomma*, *Hyalomma*, and *Rhipicephalus* ticks were found to infest the animals sampled, with *H. rufipes* being the most prevalent. Previous studies in Ghana have reported the dominance of *A. variegatum* [21–23], which is in contrast to the findings in this study. The observed difference could be due to the use of acaricides across the sampling sites, which reduced the *A. variegatum* population. Furthermore, it was observed that between the two sampled regions, the distribution of some tick species varied significantly. This could have been due to the high temperatures observed in the Upper East compared to the Greater Accra. High temperature has been reported to limit tick populations in some areas [24].

*Hyalomma* and *Amblyomma* ticks were identified to harbor CCHFV. An earlier study in Ghana with similar results detected the virus in both *Hyalomma excavatum* and *A. variegatum* ticks on slaughtered animals from a Kumasi abattoir [5]. Laboratory studies show that although *Hyalomma* spp. are well-known vectors of CCHFV, ticks of other genera that co-occur with them may also be involved in transmission [4]. Next-generation sequencing was employed to determine the specific strain of CCHFV detected in the ticks from the study area. In the sequencing process, viral enrichment was performed to increase the sensitivity of detecting CCHFV and ensure good output data for phylogenetic analysis [25].

The study found that the strain recovered in this study, T-826_GHA/2021, belongs to genotype 3 and clustered with other strains from countries including Sudan, Mali, Spain, and Mauritania. This strain data, and the finding that the CCHFV-infected ticks were collected from near the northern border in the Upper East region, suggest possible importation of the virus into Ghana. The trade movement of livestock, primarily from Burkina Faso at the northern border, likely contributes to the movement of the virus through livestock infected with CCHFV or harboring infected ticks. It is also plausible that CCHFV has been present in Ghana over the years but was not detected due to limited resources and diagnostic capabilities.

Using the S segment of the CCHFV genome, the strain in this study shared 98.17% nucleotide identity with the MF287636_Spain_2016 strain. The S segment is known to be the most conserved at the nucleotide level [26] and plays a role in the encapsulation of viral RNA by forming the ribonucleoprotein complexes [27–29] essential in assessing the topology of viruses [30].

It was observed that the M and L segments of the strain in this study shared 91.85% and 98.72% nucleotide identities with the MF689740_Spain_2018 and DQ211615_Mauritania_1984 strains, respectively. While the M segment encodes the glycoprotein precursor, the L segment encodes the RNA-dependent RNA polymerase [31]. Studies based on the three segments have shown that the CCHFV genome is prone to reassortment [32–34]. In the case of dual infection, RNA viruses with segmented genomes can reassort their segments into new genetically different viruses, which influences their pathogenicity and epidemiology [32]. However, all the segments of the CCHFV strain identified in this study clustered with other strains in genotype 3, indicating that reassortment had not occurred.

Since livestock rearing is an integral component of major communities in Ghana, there is a possible risk of zoonotic tick-borne infections, such as CCHFV, among inhabitants within these communities. While the infection rate in this initial study was low, finding CCHFV in tick vectors emphasizes the need for additional sampling, especially in the northern region. Broadened monitoring, by extending sampling to additional regions of the country and communities frequently exposed to infected ticks, as well as the blood and tissues of affected livestock, could provide important data to better inform public health strategies.

## 5. Conclusions

In this study, *H. rufipes* were found to be the dominant tick species, and we present the first whole-genome sequencing of CCHFV in this species within Ghana obtained by analyzing the S, L, and M segments. Sequence analysis indicated that CCHFV from genotype 3 (Africa 3) is present in the Upper East region (West Sudanian savanna ecological zone) of Ghana. We recommend a One-Health approach involving multiple ecological zones, the environment (biotic and abiotic factors), and blood samples from humans and livestock to further delineate the prevalence and transmission of CCHF and other tick-borne diseases within the country. This additional data is important to inform the formulation of future effective control and preventative strategies against zoonotic infections within livestock.

## Figures and Tables

**Table 1:** Distribution of tick species and CCHFV detection.

Tick species	Number of ticks, *n* (%)	CCHFV positive, *n* (%)
*Hyalomma rufipes*	290 (41.1)	2 (0.69)
*Amblyomma variegatum*	157 (22.3)	1 (0.64)
*Hyalomma truncatum*	128 (18.2)	1 (0.78)
*Rhipicephalus* spp.	71 (10.1)	0 (0.0)
*Rhipicephalus* (*Boophilus*) sp.	58 (8.2)	0 (0.0)
*Rhipicephalus sanguineus* (s.l)	1 (0.1)	0 (0.0)
Total	705	4 (0.6)

**Table 2:** Diversity of tick species between two regions in Ghana.

Tick species	Greater Accra, *n* (%)	Upper East, *n* (%)	*χ* ^2^	*df*	*p*-Value
*Hyalomma rufipes*	144 (34.9)	146 (50.0)	16.18	1	<0.001
*Amblyomma variegatum*	96 (23.2)	61 (20.9)	0.55	1	0.459
*Hyalomma truncatum*	80 (19.4)	48 (16.4)	0.99	1	0.320
*Rhipicephalus* spp.	67 (16.2)	4 (1.4)	41.67	1	<0.001
*Rhipicephalus* (*Boophilus*) sp.	26 (6.3)	32 (11.0)	4.93	1	0.036
*Rhipicephalus sanguineus* (s.l)	0 (0.0)	1 (0.3)			–
Total	413 (58.6)	292 (41.4)			

^*∗*^*p*-Value was obtained using a *χ*^2^ test.

## Data Availability

The authors confirm that all relevant data, as well as means of analysis supporting the findings of this study, are available within the article.
